# Supplementary material for: Content-rich biological network constructed by mining PubMed abstracts
Source: BMC Bioinformatics. 2004 Oct 8;5:147. doi: 10.1186/1471-2105-5-147 (PMC528731; doi:10.1186/1471-2105-5-147)
Supplement: Additional File 5 — The original Chilibot query results of the term "long-term potentiation (LTP)" and 22 other terms, limiting the latest references analyzed to the years 1990, 1995, 2000, and 2004. [file 1471-2105-5-147-S5.bz2 › chilibotAdditionalFile5/ltp2004/html/PKC_CAMKII.html]

 


 **PKC** and **CAMKII** 
  
Found 125 abstracts in PubMed,  **30 abstracts were retrieved and analyzed**.  


---

 Search Google  |
 PDF files only 
|  EDU domain only 

---

**Interactive relationship** (e.g. stimulation, inhibition, etc)

- We assume that in cholinergically stimulated parietal cells  **PKC**  alpha transinhibits  **CaMKII**  activity, resulting in an attenuation of acid secretion.  Ref: 12788814 Br J Pharmacol< MedlineTA>Br J Pharmacol, 1994
- Attenuation of  **CaMKII**  activity by  **PKC**  showed strong similarity to the downregulation of  **CaMKII**  by basal autophosphorylation.  Ref: 12788814 Br J Pharmacol< MedlineTA>Br J Pharmacol, 1994
- 6 The phosphotransferase activity of the purified  **CaMKII**  was in vitro inhibited after transphosphorylation by  **PKC**  if calmodulin was absent during transphosphorylation.  Ref: 12788814 Br J Pharmacol< MedlineTA>Br J Pharmacol, 1994
- The autonomy site of  **CaMKII**  was not transphosphorylated by  **PKC** .  Ref: 12788814 Br J Pharmacol< MedlineTA>Br J Pharmacol, 1994
- **CaMKII**  purified from parietal cell containing gastric mucosa of pig, was transphosphorylated by purified cPKC containing  **PKC**  alpha up to 1.8 mol P i per mol  **CaMKII**  in vitro.  Ref: 12788814 Br J Pharmacol< MedlineTA>Br J Pharmacol, 1994
- Together with previous studies, these results indicate that calcium influx elicited by active NMDA receptors and L VGCC triggers the Pyk2 Src signaling pathway mediated by  **PKC** , which aggravates cerebral ischemia lesions through up regulating the function of NMDA receptors after the onset of ischemia, and also could be regulated partly by CaM dependent kinases like  **CaMKII** .  Ref: 14732460 Neurosci Lett, 2004
- In the physiological conditions, Ng forms a complex with CaM, and its CaM binding affinity was modulated by phosphorylation, oxidation and glutathiolation under the activation of  **PKC**  or oxidant stress, which may be involved in the regulation of CaM and CaM activated proteins, such as CaM dependent nitric oxide synthase NOS, CaM dependent protein kinase II  **CaMKII**  and CaM dependent adenylate cyclase AC.  Ref: 12889141 Sheng Li Ke Xue Jin Zhan, 2003

**Parallel relationship** (e.g. studied together, co-existance, homology, etc.)

- We conclude that lead activates  **PKC** ,  **CaMKII**  or MEK ERK p90RSK pathways in GT1 7 cells, leading to CREB phosphorylation and modulation of gene expression.  Ref: 12832008 Brain Res Bull, 2003
- Here, we show that protein kinase C  **PKC**  and calcium calmodulin dependent kinase II  **CaMKII**  regulate the profile of human nasal CBF in response to rising temperature from 4 degrees C.  Ref: 12618214 Cell Signal, 2003
- The potentiation was inhibited by GF109203X, a selective inhibitor of protein kinase C  **PKC** ... a selective inhibitor of  **CaMKII** , or H 89, a selective inhibitor of protein kinase A PKA.  Ref: 14499485 Brain Res Mol Brain Res, 2003
- The levels of protein kinase C gamma  **PKC**  gamma and the calcium calmodulin dependent kinase II alpha  **CaMKII**  alpha were measured in crude synaptosomal P2, particulate P3, and cytosolic S3 fractions of the neocortex of rats exposed to 1 hour and 2 hour middle cerebral artery occlusion MCAO and 2 hour MCAO followed by 2 hour reperfusion.  Ref: 14688616 J Cereb Blood Flow Metab, 2004
- Onset of ciliary beat was at 10 degrees C in Medium 19 degrees C in the presence of the  **PKC**  activator phorbol 12 myristate 13 acetate PMA, the calcium ionophore ionomycin, or the  **CAMKII**  blocker myristoylated autocamtide 2 related inhibitory peptide MACI, and 6 degrees C for the myristoylated peptide  **PKC**  inhibitor EGF R Fragment 651 658 MyrPKCI.  Ref: 12618214 Cell Signal, 2003
- These results indicate that lead activated  **PKC** ,  **CaMKII**  and MEK ERK p90RSK pathways simultaneously, all of which contributed to CREB phosphorylation.  Ref: 12832008 Brain Res Bull, 2003
- In this study, we found that exposure of cortical slices to Abeta 1 42 or Abeta 25 35 induced a marked increase in the activation of protein kinase C  **PKC**  and calcium calmodulin dependent kinase II  **CaMKII** , two enzymes critically involved in a variety of cellular functions.  Ref: 12606559 J Biol Chem, 2003
- By mutagenesis experiments, we identified two phosphorylation sites of  **PKC**  and  **CaMKII**  in Kv1.4 responsible for the decrease in Ito by ET 1.  Ref: 14569155 Nippon Yakurigaku Zasshi, 2003
- 7 Our results suggest that  **PKC**  alpha and  **CaMKII**  are closely functionally linked in a cholinergically induced signalling pathway in rabbit parietal cells.  Ref: 12788814 Br J Pharmacol< MedlineTA>Br J Pharmacol, 2003
- In the present study, we provide evidence that ATP dependent activation of ERK1 2 in VSM cells utilizes specific isoforms of the multifunctional serine threonine kinases, protein kinase C  **PKC**  and calcium calmodulin dependent protein kinase II  **CaMKII**  as intermediates.  Ref: 14749212 Am J Physiol Cell PhysiolAm J Physiol Cell Physiol, 2003
- Drugs preventing  **PKC**  and or  **CaMKII**  alpha translocation may prove beneficial against ischemic cell death.  Ref: 14688616 J Cereb Blood Flow Metab, 2004
- Activation of M1 muscarinic receptors... significantly inhibited the Abeta activation of  **PKC**  and  **CaMKII** .  Ref: 12606559 J Biol Chem, 2003
- Inhibitors included bisindolylmaleimide calcium dependent and independent  **PKC** , Gö6976 calcium dependent  **PKC** , CKIINtide  **CaMKII** , KN 93  **CaMKII** , PD98059 MEK ERK1 2, and SB203580 p38.  Ref: 12826852 Anesthesiology, 2003
- To explore biochemical basis for cerebroprotective effect of immunosuppressant FK506, we studied changes in subcellular distribution of protein kinase C gamma  **PKC**  gamma as well as calcium calmodulin dependent protein kinase II  [ **CAMKII** ]   **CaMKII**  after ischemia.  Ref: 12866202 Neurol Res, 2003
- Furthermore, this action of Ang II was completely abolished by co inhibition of protein kinase C  **PKC**  and calcium calmodulin protein kinase II  **CaMKII** .  Ref: 14550259 Biochem Biophys Res Commun, 2003
- Prior studies utilizing neurons cultured from the hypothalamus and brainstem of newborn rats have demonstrated that angiotensin II Ang II induced modulation of neuronal firing involves activation of both protein kinase C  **PKC**  and calcium calmodulin dependent protein kinase II  **CaMKII** .  Ref: 12738610 Am J Physiol Regul Integr Comp PhysiolAm J Physiol Regul Integr Comp Physiol, 2003
- 5 Since both kinase modulators, TPA and KN 62, affected no divergent signal transduction pathways in the parietal cell, an in vitro model has been used to study if  **PKC**  directly targets  **CaMKII** .  Ref: 12788814 Br J Pharmacol< MedlineTA>Br J Pharmacol, 2003
- Dishevelled activates calcium flux,  **PKC** , and  **CamKII**  in vertebrate embryos.  Ref: 12771126 J Cell Biol, 2003
- In calcium clamped skinned pulmonary arterial strips from newborn rabbits, the anesthetics induce relaxation, which is prevented by the  **PKC**  inhibitors MEK ERK 12,  **CaMKII** , and p38.  Ref: 12826852 Anesthesiology, 2003
- Persistent translocations of  **PKC**  gamma and  **CaMKII**  to synaptosomal P2 fraction were observed in vehicle treated group.  Ref: 12866202 Neurol Res, 2003
- This calcium signaling acitivity is sufficient to activate calcium sensitive enzymes like protein kinase C  **PKC** , calcium calmodulin dependent kinase II  **CamKII**  or calcineurin CaCN.  Ref: 14766423 Front Biosci, 2004
- These data are the first demonstration of the phosphorylation of H 1 R by four protein kinases, i.e.,  **PKC** , PKA,  **CaMKII**  and PKG, and provide fundamental information to help us further understand the relationship between H 1 R phosphorylation and desensitization of this receptor.  Ref: 14685295 Methods Find Exp Clin Pharmacol, 2003
- The Roles of  **CaMKII** , PKA and  **PKC**  in the Induction and Maintenance of LTP of C Fiber Evoked Field Potentials in Rat Spinal Dorsal Horn.  Ref: 14586032 J NeurophysiolJ Neurophysiol, 2003
- These results suggest that Abeta activates  **PKC**  and  **CaMKII**  through enhancing excitatory activity in glutamatergic synaptic networks.  Ref: 12606559 J Biol Chem, 2003
- This Ang II mediated dipsogenic response was attenuated by central injection of the  **PKC**  inhibitors chelerythrine chloride 0.5 50 micro M, 2 micro L and Go6976 2.3 nM, 2 micro L and by the  **CaMKII**  inhibitor KN 93 10 micro M, 2 micro L.  Ref: 12738610 Am J Physiol Regul Integr Comp PhysiolAm J Physiol Regul Integr Comp Physiol, 2003
- Phorbol 12 myristate 13 acetate an activator of  **PKC** , GF109203 an inhibitor of  **PKC**  and KN 93 an inhibitor of  **CaMKII**  had no effect on AN2 promoter activity in PZHPV 7 cells.  Ref: 12899525 Recent Results Cancer Res, 2003
- In present work the roles of these three protein kinases in the induction and maintenance of LTP of C fiber evoked field potentials were evaluated by application of specific inhibitors of  **CaMKII**  KN 93 and AIP, PKA Rp CPT cAMPS and  **PKC**  chelerythrine and Go 6983 at the recording segments before and after LTP induction in Urethane anesthetized Sprague Dawley rats.  Ref: 14586032 J NeurophysiolJ Neurophysiol, 2003
- FK506 significantly decreased levels of  **PKC**  gamma and  **CaMKII**  in P2 fraction at 24 h of recovery.  Ref: 12866202 Neurol Res, 2003
- The expression patterns of cyclic AMP dependent protein kinase PKA and protein kinase C  **PKC** ... were similar to those of the forms of GluR1 phosphorylated at S845 and S831, respectively.  Ref: 12676339 Neurosci Lett, 2003
- Considerable evidences have shown that calcium calmodulin dependent protein kinase II  [ **CAMKII** ]   **CaMKII** , protein kinase A PKA and protein kinase C  **PKC**  are important for LTP in hippocampus.  Ref: 14586032 J NeurophysiolJ Neurophysiol, 2003
- By mutagenesis experiments we identified two phosphorylation sites of  **PKC**  and  **CaMKII**  in Kv1.4 responsible for the decrease in I to by ET 1.  Ref: 14521958 Biochem Biophys Res Commun, 2003
- These results suggest that activation of  **CaMKII** , PKA and  **PKC**  may be crucial for the induction and the early phase but not for the late phase maintenance of the spinal LTP.  Ref: 14586032 J NeurophysiolJ Neurophysiol, 2003
- When hm mcH 1 R, which had been immunoprecipitated with anti c myc antibody from Sf9 cell membranes, was incubated with  **PKC** , cAMP dependent protein kinase PKA, calcium calmodulin dependent protein kinase II  [ **CAMKII** ]   **CaMKII**  or cGMP dependent protein kinase PKG, the immunoprecipitated receptor was phosphorylated by these kinases.  Ref: 14685295 Methods Find Exp Clin Pharmacol, 2003
- BACKGROUND This study examined the responsiveness of skinned pulmonary arteries from newborn rabbit to volatile anesthetics and the role of protein kinase C  **PKC** , calcium calmodulin dependent protein kinase II  **CaMKII** , and the downstream effectors, mitogen activated protein kinases ERK1 2 and p38.  Ref: 12826852 Anesthesiology, 2003
- MAPK cascade interacts extensively with other protein kinases such as maturation promoting factor MPF, protein kinase A PKA, protein kinase C  **PKC** , and calmodulin dependent protein kinase II  **CaMKII**  as well as protein phosphatases in oocyte meiotic cell cycle regulation.  Ref: 14613897 Biol ReprodBiol Reprod, 2003
- **PKC**  delta and  **CaMKII**  delta 2 Mediate ATP Dependent Activation of ERK1 2 in Vascular Smooth Muscle.  Ref: 14749212 Am J Physiol Cell PhysiolAm J Physiol Cell Physiol, 2003
- We also investigated V1aR activation of calcium activated signaling kinases, protein kinase C  **PKC** , calcium calmodulin dependent protein kinase II  **CaMKII** , and the mitogen activated protein MAP kinases MAPK and extracellular signal regulated kinases 1 and 2 ERK1 2, their localization within cytoplasmic and nuclear compartments, and activation of their downstream nuclear target, the transcription factor cAMP response element binding protein CREB.  Ref: 12764111 J Neurosci, 2003
- Membrane bound hm mcH 1 R, whose conformation is closer to its physiological state than that of the immunoprecipitated receptor, was also phosphorylated by  **PKC** , PKA,  **CaMKII**  and PKG.  Ref: 14685295 Methods Find Exp Clin Pharmacol, 2003
- Co administration of calphostin C 10 microM, a  **PKC**  inhibitor and KN 93 10 microM, a  **CaMKII**  inhibitor completely blocked this Ang II action in WKY rat neurons, while they caused only a approximately 50% attenuation in SHR neurons.  Ref: 12904331 J Neurophysiol, 2003
- However, MEK1 2 inhibitor,  **CaMKII**  inhibitor, and  **PKC**  inhibitor significantly suppressed lead induced CREB phosphorylation.  Ref: 12832008 Brain Res Bull, 2003
- The authors conclude that during and after ischemia,  **PKC**  and  **CaMKII**  alpha are translocated to the cell membranes, particularly synaptic membranes, where they may modulate cellular function, such as neurotransmission, and also affect cell survival.  Ref: 14688616 J Cereb Blood Flow Metab, 2004
- Angiotensin II Ang II, acting at Ang II type 1 receptors AT1Rs, increases the firing rate of neurons from Wistar Kyoto WKY rat brain via protein kinase C  **PKC**  and calcium calmodulin kinase II  **CaMKII**  dependent mechanisms.  Ref: 12904331 J Neurophysiol, 2003
- These results demonstrate that Ang II produces an inhibition of I Kv in CATH.a cells, via an intracellular pathway that includes PLC,  **PKC** , and  **CaMKII** .  Ref: 14550259 Biochem Biophys Res Commun, 2003
- The present results suggest FK506 downregulates translocated  **PKC**  gamma and  **CaMKII** , which may contribute to its survival promoting effect after cerebral ischemia.  Ref: 12866202 Neurol Res, 2003
- Next, we utilized activators and inhibitors of protein kinase A PKA, protein kinase C  **PKC** , mitogen activated protein kinase kinase MAPKK and calcium calmodulin dependent protein kinase II  [ **CAMKII** ]   **CaMKII**  to determine which kinases might be involved in phosphorylating the CREB in PZHPV 7 cells.  Ref: 12899525 Recent Results Cancer Res, 2003
- Neither  **PKC**  inhibitors Go6983, Go6976 nor  **CaMKII**  inhibitor KN 62 had a pronounced effect on lead induced ERK1 and ERK2 phosphorylation.  Ref: 12832008 Brain Res Bull, 2003
- Results of these analyses demonstrated that V1aR activation led to a significant rise in  **PKC** ,  **CaMKII** , and ERK1 2 activation, with  **CaMKII**  and ERK1 2 demonstrating dynamic transport between cytoplasmic and nuclear compartments.  Ref: 12764111 J Neurosci, 2003
- Here we show that a Dsh deletion construct, XDshDeltaDIX, which is sufficient for activation of the PCP pathway, is also sufficient for activation of three effectors of the Wnt calcium pathway calcium flux,  **PKC** , and calcium calmodulin dependent protein kinase II  [ **CAMKII** ]   **CamKII** .  Ref: 12771126 J Cell Biol, 2003
- These experiments reveal, unexpectedly, the existence of two parallel kinase pathways, one involving  **CaMKII**  and the other PKA and  **PKC** , either of which can fully support the induction of LTP, at this stage of development.  Ref: 14529720 Neuropharmacology, 2003
- Our data suggest that  **CaMKII**  and  **PKC**  regulate the thermal slope and profile of CBF in vitro,.  Ref: 12618214 Cell Signal, 2003
- However, inhibition of  **CaMKII**  together with inhibition of either PKA or  **PKC**  fully blocked the induction of LTP.  Ref: 14529720 Neuropharmacology, 2003
